# Delivery models of HIV pre-exposure prophylaxis and their influence on uptake in South Africa: An integrative review

**DOI:** 10.4102/sajhivmed.v26i1.1684

**Published:** 2025-04-17

**Authors:** Junior M. Ntimani, Andile G. Mokoena-de Beer, Deliwe R. Phetlhu

**Affiliations:** 1 Department of Nursing Science, School of Health Care Sciences, Sefako Makgatho Health Sciences University, Pretoria, South Africa

**Keywords:** PrEP, facilitators, barriers, uptake, HIV prevention, PrEP delivery models

## Abstract

**Background:**

Maximising the use of HIV pre-exposure prophylaxis (PrEP) is crucial to eliminate new HIV transmissions, especially in high-prevalence areas such as South Africa. Strengthening access and acceptability of PrEP is essential for effective HIV prevention and to ensure sufficient uptake among those at risk.

**Objectives:**

This review aims to explore the existing PrEP delivery models in the South African public health settings and their influence on its uptake.

**Method:**

An integrative review approach was followed and electronic databases, namely PubMed, Medline, EBSCOhost, and Google Scholar, were searched. We selected qualitative and quantitative studies that focused on South Africa, written in English, and were published in peer-reviewed journals between 2016 and 2024.

**Results:**

Two distinct models were identified, namely the health facility-based model and the community-based model which is inclusive of the use of pharmacies. Both models have constraints and facilitators that impact on access and acceptability, thus influencing uptake.

**Conclusion:**

Decentralisation as a means to ensure access, and awareness to facilitate acceptability, are critical drivers of the PrEP service’s success. Therefore, it is critical to develop intervention strategies that focus on access and acceptability among the target population, driven by the need to overcome barriers and ensure sustainability.

**What this study adds:** The review emphasises that models should coexist, as they are mutually beneficial. Policymakers should recognise the importance of decentralising PrEP services and expanding delivery sites with various functionalities that meet end-user needs. This approach can enhance accessibility and effectiveness in providing PrEP services.

## Introduction

South Africa is home to the largest population of people living with HIV and also the largest antiretroviral therapy (ART) programme globally. Many people aged 15–49 years are living with HIV in South Africa, a situation that is unsustainable socially, economically, and for public health in the expansive Southern African region.^1^ In 2023, around 7.7 million people in South Africa were estimated to be living with HIV, accounting for 20% of the world’s HIV population.^2^ Of those, at least 4.7 million were receiving ART.^3^ A decreasing trend has been observed in the HIV incidence rate, although this is disproportionate depending on age and gender with more men than women experiencing these shifts.^4^ For example, since 2012, HIV incidence has decreased significantly in men aged 20–24 years (68% decrease), whereas a 44% decrease was reported among women of the same age. Adolescents aged 15–19 years, and young adults aged 20–24 years are still experiencing higher rates of new infections.^5,6^

Despite multiple behavioural awareness and biomedical interventions, reports indicate that 685 South Africans between the ages of 15- and 49-years contract HIV daily.^7^ To reduce the risk of HIV amongst adolescent girls and young women (AGYW), the WHO recommends antiretroviral (ARV) pre-exposure prophylaxis (PrEP), which clinical trials have shown to reduce HIV infection by up to 99%. Based on these findings, in July 2016, the WHO recommended the provision of PrEP for people at substantial risk of HIV infection as part of a combination HIV prevention package,^8^ which South Africa implemented in the same year.^3^

PrEP is recommended for all persons who are HIV-negative, are considered at high risk of contracting HIV, and who request it.^9^ In the South African context, PrEP is currently available as an oral dose taken once daily. The most common oral PrEP medications include: A combination of emtricitabine and tenofovir disoproxil fumarate, and a combination of emtricitabine and tenofovir alafenamide. When taken daily, oral PrEP is highly effective, reducing the risk of HIV acquisition by up to 99% from sexual transmission and at least 74% from injection drug use.^10^ To ensure that South Africa meets the HIV prevention targets, PrEP uptake and retention are key driving factors. PrEP uptake refers to both the initiation of PrEP by eligible clients and the continuation of PrEP use by those who have already enrolled in the programme.^11^ This concept encompasses two key aspects: Initiation and retention.

In South Africa, few studies have evaluated the delivery of PrEP or the service delivery models. PrEP programmes are only at the beginning of a big learning curve regarding how best to offer this new prevention service to the majority of AGYW in South Africa who are at risk of HIV.^10^ The provision of PrEP is a strategic priority for the South African Department of Health’s prevention of new HIV infections strategy.^11^ The current PrEP coverage in South Africa, as of 2023, is at 803 171 people on PrEP.^2^

The nine provinces of South Africa are responsible for providing PrEP services to the public. However, the 2019 updated report indicates that some provinces were not doing well with PrEP uptake, resulting in a negative impact on the national uptake rate.^12^ Limpopo and the Northern Cape provinces reported low numbers of PrEP initiation, at 0.1% in 2016, while KwaZulu-Natal and Mpumalanga had the highest number of people starting on PrEP, with rates of 0.5% in the same year.^12^ In the same report, the Western Cape’s uptake was less than the national average in 2016 (0.2%), 2020 (0.2%), and 2021 (0.3%). In 2021, South Africa reported an increase in PrEP uptake, reaching 1% of PrEP initiations. Nonetheless, South Africa contributed 3.3% of PrEP in 2020 to the Joint United Nations Programme on HIV/AIDS (UNAIDS) global target of 3 million.

Barriers to PrEP include stigma associated with the use of ARVs, drug side effects, frequent relocation of beneficiaries, limited resources for routine screening and medication monitoring, and a limited number of qualified healthcare workers for PrEP distribution and administration.^13^ PrEP service models aim to make PrEP more accessible and tailored to the needs of different populations. Common PrEP service models in South Africa include the facility-based and community-based models. Facility-based models are traditional models where PrEP is provided through healthcare facilities, including hospitals and clinics, while community-based models focus on providing PrEP services in community settings, such as mobile clinics, outreach programmes, and study trial sites. The facility-based model is currently the most utilised PrEP service delivery model, which includes study trial sites. However, community-based models have been implemented in some areas, but are not rolled out nationally. The facility-based model allows PrEP users to follow up and collect their medication at the facilities, whereas the community-based model requires PrEP users to collect their medication outside the clinic, most commonly through mobile clinics.

## Research methods and design

**FIGURE 1:**
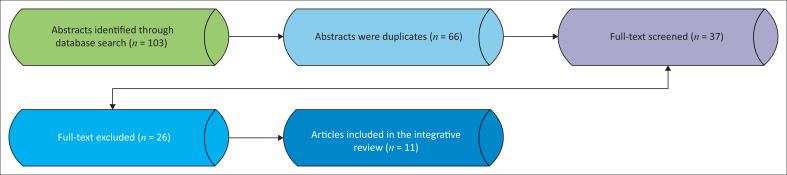
Flow chart of search strategy.

We adhered to the phases of the integrative literature review design, as outlined by Whittemore and Knafl,^14^ namely problem identification, literature search, data evaluation, data analysis, and presentation. A focused review question was formulated using the patient/population, intervention, comparison and outcomes (PICO) framework^15,16^ as follows: *What are the existing PrEP service delivery models and their influence on PrEP uptake?* Google Scholar, Medline, PubMed, and EBSCO host were used as databases for article search. We combined terms and statements PrEP *and* implementation *or* service delivery models, PrEP *and* uptake, and intervention *or* strategies. The Medical Subject Headings (MeSH) engine was employed to search the database vocabularies for additional terms. The selection process involved reading abstracts of each study identified according to the inclusion criteria, namely qualitative, and quantitative peer-reviewed studies written in English, published from 2016 to 2024, and focusing on nurses’ PrEP implementation and uptake. The initial search identified 103 articles; however, 66 were duplicates (Figure 1). Of the 37 full texts read, 26 were excluded as being low quality because they scored below 6 according to the Quality Assessment of HIV/AIDS Provider Training Tool (QAHPTT) critical appraisal checklist, which sets good quality at a score of 7–10. Finally, 11 articles were included for data extraction (Figure 1), which focused on the study setting, study design, sample, methods, and outcomes of the study.

Every article that was included was read several times by each of the three authors and any patterns that were found were coded. To better perform comparisons across all included studies and spot trends, a matrix was used to display the data using the qualitative content analysis method.^17,18^ The codes were then combined and compared to create the themes and sub-themes outlined in Table 1 as per consensus among all authors.

**TABLE 1:** Identified themes and sub-themes.

Theme	Sub-themes
1. Service delivery models	1.1. Facility-based model 1.2. Community-based model
2. Barriers to PrEP uptake	2.1. Patient-related barriers 2.2. Facility-related barriers 2.3. Community-related barriers
3. Facilitators of PrEP uptake	3.1. Personal motivation, risk perception and relationships dynamics 3.2. Disclosure, social support and PrEP counselling 3.3. Healthcare access and integrated health services delivery
4. Integration gaps	4.1. Resource limitations
5. Opportunities	5.1. Expansion of community services 5.2. Introduction of innovative services

PrEP, pre-exposure prophylaxis.

### Ethical considerations

This study was approved by Sefako Makgatho University Research Ethics Committee (reference number: SMUREC/H/485/2023:PG). The study did not involve any participants, thus informed consent was not obtained. The study was conducted in accordance with the principles of honesty and transparency in the application of the methodology’s phases and accurate data reporting as outlined by Wager and Wiffen.^19^

## Results

### Study demographics

The 11 included articles reported on 4326 individuals, which included predominantly women from the AGYW population, pregnant and postpartum women, female sex workers (FSWs), peer educators, and healthcare workers (Table 2). Seven of the 11 studies used a qualitative approach, three were quantitative and one was mixed method in nature. Included studies were conducted in Buffalo City, Cape Town, Durban, Polokwane, and Pretoria representing, five of the nine South African provinces.

**TABLE 2:** Characteristics and summary of articles included in the review.

Author and year	Reference no.	Setting	Research design	Method	Outcome
Beesham et al., 2023	20	KwaZulu-Natal (Durban)	Qualitative	**Sample:** *N* = 13 female participants **Data collection:** In-depth telephonic interviews **Analysis:** Inductive thematic analysis	**Model**: Research clinic and/or study trial site (community-based). **Uptake:** 100% (*N* = 13) initiations, six out of seven accessed PrEP post study trial; however five later discontinued. The remaining seven did not have access to PrEP. **Barriers:** Side effects; change of access site; unavailable treatment; fear of stigma; not in the collection site area; long waiting times; inconvenient operating hours; locations far from women’s homes; work commitments; inability to afford transportation to collect site; provider-related factors such as confidentiality; lack of referral system. **Facilitators:** Condom-related; partner-related; prevent and/or protect oneself from acquiring HIV and/or to feel safe; gender stereotypes; risky sexual behaviour; planning for pregnancy; positive reactions and/or supported PrEP use by those to whom use is disclosed; personal strategies to continue using PrEP; use of referral letter; distance to the collection site; transportation to the new site. **Opportunities:** Transport services; increasing knowledge and awareness on oral PrEP among oral PrEP users, their family members, communities, and healthcare providers; readily available PrEP at all clinics; more convenient clinic operating hours, including weekends. The integration of PrEP delivery with other services, such as pharmacies, the provision of longer-acting PrEP methods, and the provision of oral PrEP in a trial and/or research setting were also suggested.
Davey et al., 2022	21	Western Cape (Cape Town)	Quantitative	**Sample:** *N* = 1201 pregnant **Data collection:** Survey, face-to-face interviews **Analysis:** Frequencies and percentages for categorical variables, and median and interquartile range (IQR) for continuous variables; univariate and multivariable Poisson regression models for prevalence ratios and adjusted prevalence ratios	**Model:** Clinic-based model. **Uptake:** 84% of women initiated PrEP during the first antenatal care (ANC) visit (*n* = 1014). Women in their first pregnancies initiated PrEP less than women with prior pregnancies (72% vs 85%; *P* = 0.05). Women diagnosed and treated same day for an STI in ANC were more likely to begin PrEP at initiation compared to women without an STI (93% vs. 84%; *p* < 0.001); Pregnant women who experienced IPV in the past year also initiated PrEP more than those who did not experience IPV (89% vs. 84%, *p* = 0.09); continuation is not related to age, education, or relationship status. **Barriers:** Side effects, pill burden associated with daily PrEP intake, low risk perception. **Facilitators:** PrEP counselling; previous IPV; history of alcohol and drugs use; no side effects experienced; earlier in their pregnancy; HIV-positive partner; access to PrEP in real time. **Opportunities:** PrEP integration into maternal childcare and paediatric care; non-blanket approach allowing choice regarding long-or short-acting drugs; decentralised services to community services for postpartum, breastfeeding women.
Makhakhe et al., 2022	22	KwaZulu-Natal (Durban)	Qualitative	**Sample:** *N* = 39: 11 peer educators, 2 health workers, and 26 female sex workers **Data collection:** In-depth interviews and focus group discussions **Analysis:** Inductive thematic analysis	**Model:** Clinic-based model. **Barriers:** Lack of distinction between PrEP and ARVs; PrEP being associated with high-risk groups, stigma and lack of clear messaging and education from healthcare providers. Widespread misconception about sex workers being HIV-positive. The focus on distributing PrEP only to FSWs, exclusion of other women at risk. **Facilitators:** Peer education; different service delivery points for PrEP and SRH services (healthcare facilities, pharmacies, schools, universities, police stations, pubs, eateries, parks, etc.). **Opportunities:** Extend PrEP to the general population; implement measures to reduce stigma. Improve coordination and training between PrEP distribution organisations and public health facilities; provide proper education about PrEP. Provide more support and education for FSWs; explain PrEP use to partners; address misconceptions about HIV prevalence. Balance support between HIV-negative and HIV-positive FSWs taking PrEP/ARVs.
Mataboge et al., 2023	23	Gauteng (Pretoria)	Qualitative	**Sample:** *N* = 109 AGYW, pregnant AGYW, FSWs, adolescent boys, and young men, and MSM **Data collection:** Focus group discussions using semi-structured interview guide **Data analysis:** Inductive thematic analysis	**Model:** Clinic-based model. **Uptake:** Regarding PrEP use, just over a third of all participants (33.9%) had ever used PrEP; higher among women (38.0%) than men (29.6%). PrEP was predominantly collected from a public health clinic (59.5%), followed by mobile outreach services (35.1%), and from private projects/organisations (2.7%). **Barriers:** Concerns about side effects. **Facilitators:** Disseminating health information and engagement; friendly staff. **Opportunities:** Simplified PrEP service delivery and new long-acting HIV prevention methods. Decentralise and simplify PrEP; provide SRH service for men outside traditional healthcare settings. Tailor service delivery options according to needs and preferences of different population groups. Provide a variety of long-acting PrEP methods. Address concerns about the Dapivirine Vaginal Ring through counselling, sex-positive messaging, and engagement with potential users, partners, and other key stakeholders.
Moran et al., 2022	24	Western Cape (Cape Town)	Quantitative	**Sample:** *N* = 623) pregnant women **Data collection:** Survey, face-to-face interviews **Data analysis:** Statistical analyses	**Model:** Research clinic and/or study trial site (community-based). **Uptake:** 91% (*n* = 570) of *N* = 623 pregnant women initiated on PrEP at baseline. **Barriers:** In the same year, the country adopted the use of PrEP as an HIV preventative strategy for individuals who were at high risk of acquiring HIV in addition to the existing prevention strategies. **Facilitators:** PrEP counselling and maternal PrEP interventions. **Opportunities:** Improved awareness building among PrEP users; target the broader community to improve overall knowledge of PrEP and its benefits.
Pillay et al., 2020	25	Limpopo, Gauteng, KwaZulu-Natal, Western Cape	Mixed method	**Sample:** *N* = 299 (*n* = 203 from sex worker facilities, *n* = 96 from MSM facilities) **Data collection:** Surveys and in-depth interviews **Data analysis:** STATA 13 (StataCorp LLC, College Station, Texas, United States) for survey data and thematic analysis using NViVO 11 (QRS International, Melbourne, Australia)	**Model:** Clinic-based model. **Uptake:** 8% for sex workers in a clinic-based model in Limpopo. 8% for sex workers, 35% of MSM in a clinic-based model and 22% community-based model in Gauteng for sex workers. 10% in a community-based model and 49% in a clinic-based model for sex workers in KwaZulu-Natal. 13% in a clinic-based model for MSM in the Western Cape. **Barriers:** Fear of side effects and stigma related to HIV. **Facilitators:** Perceived risk associated with sexual activity. **Opportunities:** Provide valuable insight on how clients perceive PrEP; direct efforts to improving acceptance of PrEP; develop well-designed, effective, and brief information, education and communication (IEC) materials; seek user feedback.
Pleaner et al., 2022	26	Gauteng, KwaZulu-Natal, Eastern Cape	Qualitative	**Sample:** *N* = 48 healthcare workers **Data collection:** In-depth face-to-face and telephonic interviews **Data analysis:** Descriptive analysis was used on demographic data. Qualitative data used NVIvo software (version 12, QRS International, Melbourne, Australia) using inductive coding approach	**Model:** Facility- and community-based model. The Project Mobile Clinic provided youth with access to healthcare at secondary and tertiary educational institutions. **Barriers:** Overburdened health system associated with integration of PrEP service, diversion of resources from critical services and limited resources and time. **Facilitators:** Supermarket approach; reduced waiting time. Provide opportunities for other health services managing STI; promote dual methods and avert unplanned pregnancies. **Opportunities:** Strengthen the provision of adolescent- and youth-friendly services, include adolescent-health provider dialogues in the clinics. Extend clinic-based model PrEP services to the surrounding community.
Rousseau et al., 2021	27	Western Cape (Cape Town)	Qualitative	**Sample:** *N* = 30 women **Data collection:** In-depth interviews **Data analysis:** Inductive thematic analysis.	**Model:** Community-based (Tutu Teen Truck [TTT]) mobile health clinic. Provide services in locations and at times that build on where young women organically network, such as schools, community centres, and public transport hubs. **Uptake:** 32.7% AGYW initiated on PrEP on the same day. **Barriers:** Community riots, violence, and severe weather conditions. **Facilitators:** Convenient community locations and high visibility. Eliminate the cost of transport, and an easy appointment system. **Opportunities:** Encourage integration of SRH services; offer a holistic prevention approach to AGYW sexual health. Provide health education on HIV prevention and contraception to AGYW.
Shamu et al., 2021	28	Gauteng	Quantitative	**Sample:** *N* = 1917 HIV-negative women and men **Data collection:** Household survey **Data analysis:** Stata 13.0	**Model:** Community-based model (home visits). Provide HIV education, minimise unsafe sexual practices, and promote HIV testing and treatment in communities. **Uptake:** 49% were keen to initiate PrEP; over 51% had no knowledge of PrEP. **Barriers:** Inadequate PrEP knowledge, dislike taking pills daily and side effects. **Facilitators:** Having tuberculosis and PrEP knowledge. **Opportunities:** Promote youth’s PrEP awareness, incorporate a multifarious media strategy.
Smith et al., 2023	29	Eastern Cape (Buffalo City)	Qualitative	**Sample:** *N* = 22 men **Data collection:** In-depth interviews **Data analysis:** Inductive thematic analysis	**Model:** Community-based (mobile gazebo used in the Community PrEP Study). Utilise community-based platforms to assist young women access and adhere to PrEP more frequently. **Uptake:** 100% (*N* = 22) initiated on PrEP. **Barriers:** Compulsory HIV testing for men. **Facilitators:** High risk of HIV acquisition associated with alcohol use and unprotected sex with multiple partners; quick access to PrEP services. **Opportunities:** simplified access to PrEP service model; include delivery or pharmacy pickup services during working hours.
Wyatt et al., 2023	30	KwaZulu-Natal (eThekwini)	Qualitative	**Sample:** *N* = 25 women **Data collection:** In-depth interviews **Data analysis:** A matrix approach	**Model:** Research clinic and/or study trial site (community-based). **Uptake:** 76% (*n* = 19 of 25) of women who were planning to be pregnant in the next 12 months initiated on PrEP. **Barriers:** Changing proximity to male partners; COVID-19 lockdown; mobile lifestyle; PrEP-related stigma; and disclosure of PrEP use. **Facilitators:** Not living with partners; partners as drivers of pregnancy intention; and feeling at high risk for HIV. **Opportunities:** Following HIV and safer conception counselling; women chose to postpone motherhood. Male partners were perceived as the primary sources of reproductive desire in the relationship.

Note: Please see the full reference list of the article, Ntimani JM, Mokoena-de Beer AG, Phetlhu DR. Delivery models of PrEP and their influence on uptake in South Africa: An integrative review. S Afr J HIV Med. 2025;26(1), a1684. https://doi.org/10.4102/sajhivmed.v26i1.1684, for more information.

ARVs, antiretrovirals; ANC, antenatal care; AGYW, adolescent girls and young women; FSW, female sex workers; IEC, information, education and communication; IPV, intimate partner violence; MSM, men who have sex with men; PrEP, pre-exposure prophylaxis; STI, sexually transmitted infection; SRH, sexual and reproductive health.

### Key themes

The analysis yielded five main themes on the PrEP delivery models and their influence on PrEP uptake in South Africa, namely service delivery models, barriers to PrEP uptake, facilitators of PrEP uptake, integration gaps, and opportunities (Table 1).

#### Theme 1: Service delivery models

Two primary models (facility-based and community-based) were identified. The clinics and hospitals offer a facility-based model through the ART programme. PrEP users are given review or follow-up dates, which include monitoring of vital data, blood tests and medication collection. The patient’s actual day of visit and return dates are recorded in the facility’s TIER.net capturing system, which is used for monthly facility reports. Community-based models are offered in the community through the mobile clinics and study trial sites, thus integrating PrEP distribution within comprehensive sexual and reproductive healthcare (SRH) services via a community-based model, particularly with mobile clinics, providing various advantages. By providing PrEP alongside other SRH services, clients obtain a more comprehensive approach to their health, addressing various needs on a single visit. In addition, mobile clinics deliver services directly to the community, allowing people to receive care without having to travel long distances. Mobile clinics can target high-risk locations and communities, ensuring that individuals who require PrEP and other SRH services obtain them. Providers can provide individualised education and support to help clients understand the value of PrEP and how to use it effectively.

**Sub-theme 1.1: Facility-based model:** This review included four articles that identified facility-based models.^20,22,24,25^ The facility-based model for providing PrEP and SRH services was identified as a potential driver of PrEP acceptance. This model is available in South Africa’s primary healthcare facilities through the Department of Health. Healthcare professionals who have received PrEP and ART guidelines training provide the service. The service is not a stand-alone, but rather falls under the ART programme, and most facilities are supported by non-governmental organisations (NGOs) such as the Anova Health Institute. Although there are challenges with this model, this model is currently recruiting more patients in the system because it is used in all facilities that are rendering PrEP service.

**Sub-theme 1.2: Community-based model:** This review included four articles that identified the need for decentralisation of PrEP service delivery.^20,22,24,25^ This review has revealed that prospective PrEP users are keen to utilise PrEP services offered from the community’s different locations. An NGO provides the service, with healthcare professionals trained in ART and PrEP guidelines, and is funded by private agencies such as the President’s Emergency Plan for AIDS Relief. Although the targeted population expressed desire to use a community-based model as their PrEP service delivery, this model encounters challenges that cause the interruption of services, such as community riots, violence, and severe weather.^24^ These specific groups prefer community-based models rather than facility-based models because of patient- and facility-related barriers, such as long waiting times and facility operating times. However, the issue around the safety and security of users was highlighted as a concern. The use of PrEP service delivery sites such as police stations was met with mixed reactions, with some AGYW stating that the element of privacy would be compromised.^20,22^ This model of delivery is currently not recruiting the majority of users into the system, as it is not implemented country wide.

#### Theme 2: Barriers to pre-exposure prophylaxis uptake

The review identified barriers to PrEP uptake as being patient-related, facility-related, and community-related. Patient-related barriers are challenges that affect patients on an individual level, whereas facility-related barriers are challenges encounter by PrEP users in the clinic, and community-related barriers are challenges faced by PrEP users and services providers in the community.

**Sub-theme 2.1: Patient-related barriers:** Barriers to PrEP uptake were identified in four of the seven articles reviewed.^20,21,22,25^ Another review reported that men identified HIV testing prior to PrEP initiation as a barrier to PrEP uptake, as they were concerned about testing HIV-positive after having unprotected sex.^25^ The implications of PrEP side effects could contribute to poor PrEP uptake. Barriers related to PrEP medication (tenofovir, emtricitabine) as part of ARVs could mislead potential PrEP users. This exacerbated the fear of stigma and raised doubts among those who want to use PrEP because it is associated with ARVs, which are stigmatised.^21^ All of the barriers mentioned were applicable to both the facility- and community-based models.

**Sub-theme 2.2: Facility-related barriers:** Two articles included in this review identified facility-related barriers.^20,26^ Clinic attendance remains critical for PrEP users as long as they are at risk of acquiring HIV. One of the studies included in this review found that individuals who enrolled through the study trial site complained of long waiting times, inconvenient operating hours, locations far from PrEP users’ homes, and transport costs to collect PrEP because the clinics were far from where they lived after the study trial.^20^ As the programme’s implementers and drivers, the attitudes of clinic employees, including nurses, HIV Testing Services counsellors, and administrative clerks, have a significant impact on whether the programme succeeds or fails. Furthermore, this may have a negative impact on the retention of current PrEP service users, resulting in a high defaulter rate.^20^ This review highlighted that women encountered PrEP medication shortages and were turned away without medication. These barriers demonstrate that more work needs to be done at a faster pace to accomplish the global goal of eliminating new HIV infections by 2030. The UNAIDS goal of zero new infections may not be achieved unless a few changes are made regarding ART and the PrEP programme in local facilities.

**Sub-theme 2.3: Community-related barriers:** Although community-based mobile clinics are an innovative approach to offering PrEP services to patients, they have certain drawbacks. One article in this review highlighted the challenges that the community-based model, and the mobile clinic in particular, encounters, with issues including organising the move and setting up of mobile clinics, and bad weather which can cause service interruptions, making continuous access impossible.^27^ According to the review, challenges such as violence and community riots make it difficult to provide residents with daily services. Moreover, violence and unrest in the community might put staff and patients in danger, which can cause service interruptions.

The stigma associated with visiting mobile clinics for PrEP, which is occasionally mistaken with HIV-positive treatment, may prevent some people from utilising these services. Maintaining mobile clinic operations necessitates ongoing funding, which can be difficult to obtain, as well as hiring and maintaining trained healthcare providers prepared to provide care in mobile clinics. On the contrary, integrating mobile clinic services into current healthcare systems and ensuring continuity of care could be challenging because of the resources required to run these services. To keep accurate and secure patient information in mobile clinics, robust data management systems are required. Despite these challenges, mobile clinics remain an effective strategy for enhancing PrEP access, particularly in poor and high-risk areas. Addressing these challenges entails strengthening logistical planning, obtaining long-term funding, increasing community engagement, and better integrating services with the larger healthcare system.

#### Theme 3: Facilitators of pre-exposure prophylaxis uptake

Facilitators of PrEP uptake were identified in three articles included in this review, discussed under the sub-themes below.

**Sub-theme 3.1: Personal motivation, risk perception and relationship s dynamics:** According to the articles analysed, PrEP users have a personal motive to stay HIV-negative. PrEP users who were unaware of their partner’s HIV status felt compelled to initiate PrEP so that they could avoid contracting HIV in case their partners were HIV-positive. Five of the analysed articles identified that most PrEP users were uninformed of their partners’ HIV statuses.^20,21,25,29,30^ PrEP provides people with a sense of control over their sexual health and minimises anxiety about HIV. The desire to avoid HIV infection is an effective facilitator, especially for people who believe they are at high risk of infection. PrEP users realised they needed to take PrEP as an HIV prevention method after acknowledging that they engaged in risky behaviours such as drugs and alcohol use.

**Sub-theme 3.2: Disclosure, social support and pre-exposure prophylaxis counselling:** Disclosure, social support, and PrEP counselling are all critical components of effective PrEP use. Openness and disclosing PrEP use with partners, family and friends can lead to better support and understanding, hence improving adherence. Four articles included in this review highlighted disclosure, social support, and PrEP counselling as critical components of PrEP uptake.^22,23,24,28^ While challenges such as fear of stigma or negative responses can make disclosure difficult, they may have a detrimental impact on user adherence. A solid support network, which includes supportive partners, family and friends, can help people adhere to PrEP more effectively.

Community-based support groups and peer networks can offer encouragement and useful information, making it easier to stick with PrEP by removing social barriers. Furthermore, PrEP counselling provides critical information regarding PrEP, such as its advantages, potential side effects, and the need of adherence. Counsellors can assist people in navigating personal problems, increasing motivation, and developing techniques for sticking to their goals. Tailored interventions, such as personalised therapy sessions, can address individual concerns and challenges to PrEP use, making it easier and more successful. These components work together to provide a supportive environment that encourages individuals to both start and continue using PrEP effectively.

**Sub-theme 3.3: Healthcare access and integrated health services delivery:** Healthcare access and integrated healthcare delivery are critical for optimal PrEP acceptance and adherence including uptake. Four articles included in this review indicated healthcare access and integrated health service delivery as critical components of promoting PrEP use.^26,27,28,29^ Making PrEP available in a variety of healthcare settings, such as clinics, hospitals, and mobile clinics, improves accessibility for a wide range of potential users. One article in this review revealed that men preferred a community-based model as their PrEP service delivery model.^29^ Expanding PrEP services to remote and underprivileged areas via mobile clinics can help to address access barriers. Furthermore, combining PrEP treatments with other healthcare services, such as sexual health clinics, maternity and childcare, allows for a more complete approach to patient care. While streamlining referral and follow-up processes within healthcare systems can increase the efficiency and effectiveness of PrEP distribution. Adapting PrEP treatments to the unique needs of different groups, particularly individuals with co-occurring health concerns, improves the entire healthcare experience. These methods contribute to the development of a supportive atmosphere that helps people initiate and remain on PrEP.

#### Theme 4: Integration gaps

Integration gaps were identified as a hindrance to the delivery of PrEP service.

**Sub-theme 4.1: Resource limitations:** Three articles included in this review mentioned the integration of PrEP services.^20,24,26^ Limited resources can have a substantial impact on the uptake and effectiveness of PrEP treatments. Overburdened healthcare facilities may be unable to provide efficient PrEP medications, particularly because of a shortage of qualified healthcare workers. Furthermore, inadequate funding for PrEP programmes may limit the availability of drugs and support services. The lack of linked electronic health record systems can make it difficult to identify and manage patients who are eligible for PrEP. Access to complete healthcare services remains crucial for incorporating clinical service management as a component of the ideal clinic. However, lack of resources may deter this objective. This review reveals that healthcare providers were concerned about the addition of another service to an already overcrowded health system, particularly the ART programme.^26^ Furthermore, most healthcare providers recognised the benefits and opportunities provided by PrEP and SRH service integration. However, providers highlighted the need for additional human resources to assist in carrying the workload.

#### Theme 5: Opportunities

Opportunities for expansion and introduction of innovative services were identified in this review as opportunities for improving the delivery of PrEP services.

**Sub-theme 5.1: Expansion of community services:** This review identified articles that suggested the need for extension of clinic-based model to community-based model.^20,22,23,25^ The findings from this review underscore the critical importance of growing the PrEP service delivery mode to meet the needs of diverse populations, particularly key populations. Thus, necessitating the expansion of PrEP delivery with other services such as pharmacies, mobile clinic and school health, would increase the acceptability of PrEP amongst the key population. The provision of longer-acting PrEP methods to reduce frequent clinic visits, and the provision of oral PrEP in a trial and/or research setting, were also suggested as possible facilitators of PrEP uptake. Additionally, there is a need for the PrEP service delivery models to be transformed. Hence, Beesham and colleagues^20^ emphasised a transition from a clinic-based model to a community-based PrEP service delivery model to eliminate structural barriers to access.

**Sub-theme 5.2: Introduction of innovative services:** This review identified and emphasised the need for introduction of innovative services,^20,22,27,28^ thereby suggesting the need for paradigm shift from traditional clinic-based models toward more accessible, user-centred approaches. Such a shift recognises that a one-size-fits-all approach is insufficient for achieving optimal PrEP coverage across diverse population groups. Accordingly, decentralising and simplifying PrEP and SRH service delivery by providing services outside of traditional healthcare settings can increase access. Tailoring service delivery options to fit the needs and preferences of different population groups is integral to improve access. Providing a variety of long-acting PrEP methods to increase access, uptake, and continuation, and to decrease new HIV infections while addressing concerns about the Dapivirine Vaginal Ring through counselling, sex-positive messaging, and engagement with potential users, partners, and other key stakeholders, can improve acceptability and uptake.

## Discussion

This integrative review explored the existing PrEP delivery models in South African public health settings and their influence on PrEP uptake. The findings reveal a complex interplay of factors affecting PrEP implementation and utilisation, highlighting both challenges and opportunities for improving HIV prevention strategies in the country.

### Delivery models and their impact

Different PrEP delivery models have varying impacts on the uptake and adherence to PrEP. Two primary PrEP delivery models were identified: facility-based and community-based models.

Traditional facility-based models provide a structured environment for PrEP delivery, ensuring comprehensive care and follow-up. However, they may be less accessible to individuals in remote or underserved areas. Integrating PrEP with other healthcare services, such as sexual health clinics and harm reduction programmes, ensures a holistic approach to patient care. This model can improve adherence by addressing multiple health needs simultaneously.

Community-based models, including mobile clinics, bring PrEP closer to high-risk populations, increasing accessibility and convenience. These models can significantly boost uptake, especially in areas with limited healthcare infrastructure. This review suggests that the community-based model, such as the use of mobile clinics, may enhance PrEP uptake because it offers significant advantages in terms of accessibility and acceptability, particularly for key populations such as AGYW, men who have sex with men (MSM), and FSWs. The preference for community-based models among certain groups, notably men and young people, stems from the perceived barriers associated with facility-based model, including long waiting times, inconvenient operating hours, and concerns about privacy and stigma. This aligns with global trends in HIV prevention, which increasingly emphasise the importance of differentiated service delivery to meet the diverse needs of at-risk populations.^28^

### Barriers to pre-exposure prophylaxis uptake

Several barriers to the uptake of PrEP have been identified, and can be categorised into structural, social, clinical, and behavioural factors. Limited availability of PrEP services in certain areas, especially rural and underserved regions, can hinder access to PrEP services. Clinic-based barriers, such as long waiting times, inconvenient locations, and medication shortages, underscore the systemic challenges within South Africa’s healthcare system. Stigma associated with HIV and PrEP use can deter individuals from seeking PrEP. This includes stigma from healthcare providers, family, and the community. Patient-related barriers include concerns about HIV testing, fear of side effects, and stigma associated with ARVs. These findings echo broader challenges in HIV prevention, where stigma and misconceptions continue to hinder service utilisation.^31^ Some healthcare providers may lack sufficient knowledge about PrEP, leading to missed opportunities for prescribing it to at-risk individuals. Individuals who do not perceive themselves to be at high risk for HIV may be less likely to seek out PrEP. Challenges with adhering to a daily medication regimen can impact the effectiveness of PrEP and discourage its use. Addressing these barriers requires a multifaceted approach, including increasing awareness and education, reducing stigma, improving healthcare provider training, and making PrEP more accessible and affordable.

### Facilitators and opportunities

Despite these challenges, the review also highlighted several facilitators of PrEP uptake. The desire to remain HIV-negative emerged as a strong motivator, particularly among high-risk groups. This suggests that targeted education and awareness campaigns emphasising the efficacy of PrEP could be effective in increasing uptake. The integration of PrEP services with SRH services, especially in ANC settings, shows promise in increasing PrEP initiation among pregnant women. However, the significant drop in PrEP continuation post delivery indicates a need for better transitional care and follow-up mechanisms.

### Integration and resource constraints

The review underscores the complexities of integrating PrEP services into existing healthcare frameworks. While integration offers potential benefits in terms of comprehensive care and efficiency, it also places additional strain on an already overburdened health system. The concerns raised by healthcare providers about resource allocation and workload highlight the need for careful planning and adequate resourcing of PrEP programmes.

### Limitations and future research

The scope of the review was restricted to South Africa and the limited number of studies included in this review restricted the conclusions drawn. This has narrowed the reviews from other countries; thus, future research should aim to provide a more comprehensive national picture of PrEP implementation and uptake. Additionally, longitudinal studies examining the long-term impact of different delivery models on PrEP adherence and HIV incidence would be valuable.

### Implications for policy and practice

The findings below have several implications for PrEP implementation in South Africa.

#### Diversification of delivery models

There is a clear need to expand community-based PrEP delivery options, including mobile clinics, pharmacy-based services, and integration with other community health initiatives. This could help overcome many of the facility-related barriers identified.

#### Targeted interventions

Tailoring PrEP services to the specific needs and preferences of different population groups (e.g. AGYW, MSM, FSWs) could improve uptake and retention. This may include offering PrEP in youth-friendly settings or integrating services with existing sexual health programmes.

#### Addressing stigma

Comprehensive strategies to reduce HIV-related stigma, both in healthcare settings and communities, are crucial for improving PrEP uptake and adherence.

#### Strengthening health systems

Addressing facility-related barriers requires broader health system strengthening, including improving supply chain management, increasing human resources, and enhancing clinic efficiency.

#### Continuity of care

Developing strategies to improve PrEP continuation, particularly during transitions (e.g. the postpartum period), is essential for maximising the impact of PrEP programmes.

#### Resource allocation

Policymakers need to consider the resource implications of PrEP integration carefully, ensuring that implementation does not come at the expense of other critical health services.

## Conclusion

The successful implementation of PrEP in South Africa requires a nuanced approach that addresses both structural and individual barriers to uptake. While the current facility-based model provides a foundation for PrEP delivery, the expansion of community-based and integrated services offers promising avenues for increasing access and acceptability. As South Africa continues to grapple with a high HIV burden, optimising PrEP delivery models will be crucial in realising the potential of this powerful prevention tool and moving closer to the goal of eliminating new HIV infections by 2030.
